# Male × Female Interaction for a Pre-Copulatory Trait, but Not a Post-Copulatory Trait, among Cosmopolitan Populations of *Drosophila melanogaster*

**DOI:** 10.1371/journal.pone.0031683

**Published:** 2012-03-14

**Authors:** Alison Pischedda, Andrew D. Stewart, Monica K. Little

**Affiliations:** Department of Ecology, Evolution and Marine Biology, University of California Santa Barbara, Santa Barbara, California, United States of America; University of Arkanas, United States of America

## Abstract

Sexual coevolution occurs when changes in the phenotype of one sex select for changes in the other sex. We can identify the “footprint” of this coevolution by mating males and females from different populations and testing for a male-female genotype interaction for a trait associated with male (or female) performance. Here we mated male *Drosophila melanogaster* from five different continents with females from their own and different continents to test for a male-female interaction for mating speed, a pre-copulatory trait, and female reproductive investment, a post-copulatory trait. We found a strong male-female interaction for mating speed, consistent with previous studies using different populations, suggesting that the potential for sexual coevolution for this trait is present in this species. In contrast, we did not detect a male-female interaction for female reproductive investment. Although a male-female interaction for mating speed is compatible with the hypothesis of ongoing sexual coevolution, the nature of our experimental design is unable to exclude alternate explanations. Thus, the evolutionary mechanisms promoting male-female genotype interactions for pre-copulatory mating traits in *D. melanogaster* warrant further investigation.

## Introduction

Males and females interact in a wide diversity of ways in the context of reproduction, and these interactions provide many opportunities for the sexes to coevolve. Much of this coevolution is expected to be driven by sexual selection and lead to complementary phenotypes, such as species-specific pheromones and their receptors [1] or congruence in the shape of genitalia [2]. Because sexual selection can rapidly affect sexual traits within populations, it has the potential to generate rapid divergence and reproductive isolation between populations [3]. Intersexual coevolution can also be antagonistic when sexual conflict exists in a population. If the optimal outcome of male-female interactions differs between the two sexes, sexual conflict can favor increased fitness in one sex at a cost to the other, thus selecting for counter-adaptations in the harmed sex to mitigate these costs [4], [5]. As with coevolution driven by sexual selection, this coevolutionary “arms race” between the sexes has the potential to evolve very rapidly within populations [6], and as such, has also been implicated in population divergence and speciation [7]–[9].

We can identify the “footprint” of intersexual coevolution by mating males and females from different populations and measuring variation in a trait associated with male (or female) performance [10]. If male performance changes depending on the genotype of his mate (as indicated by a male-female genotype interaction), this suggests that evolution of the female trait has the potential to lead to coevolutionary changes in the male trait (and vice versa). Although a male-by-female interaction indicates the potential for coevolution to operate between the sexes, it does not definitively demonstrate that this process is occurring, nor can it reliably distinguish between coevolution caused by mutually beneficial or antagonistic processes [11].

Here, we focus on specific pre-copulatory and post-copulatory traits that both have the potential to result in sexual coevolution. Mating speed, the time to achieve copulation by a specific mating pair, is a pre-copulatory trait indicating female (and possibly male) mating preferences (with the assumption that “preferred” males generally have shorter mating speeds; [12]). Previous studies have shown genetic variation for mating speed among male and female *D. melanogaster* within populations [13], and there is evidence that mating preferences can differ between *D. melanogaster* populations as well. For example, females collected from Zimbabwe strongly prefer to mate with males from their own population over males from other cosmopolitan populations, whereas cosmopolitan females generally show little to no aversion against Zimbabwe males [14]. This pattern is consistent with pre-copulatory intersexual coevolution within the Zimbabwe population, potentially operating via a Fisherian runaway process [15].

We recently identified a new post-copulatory trait with the potential to result in intersexual coevolution in *D. melanogaster*: the amount of female investment in broods produced soon after mating [16]. For example, females may assess male quality using sexually selected traits and adjust their investment in reproduction accordingly [17], or males may stimulate females to invest heavily in broods produced soon after mating, when paternity confidence is often the highest. In a previous study, we found substantial genetic variation within populations in male ability to influence short-term female reproductive investment (measured by incorporating both fecundity and egg size) using 50 isofemale lines originally collected from 5 distant populations of *D. melanogaster*: 10 lines each were surveyed from populations in Ithaca (New York), Beijing (China), Tasmania, the Netherlands and Zimbabwe [16]. However, this male influence was identified using a single female genotype. To measure the opportunity for male-female coevolution, we needed to screen additional female genotypes to test whether a male-female interaction exists.

Here, we selected a single isofemale line from each of the five populations of *D. melanogaster* we previously surveyed [16]: Ithaca, Beijing, Tasmania, the Netherlands and Zimbabwe. We mated the males from these five isofemale lines to females from the same five isofemale lines in all possible combinations and measured mating speed and female reproductive investment for each combination to test for a male-female interaction. Any such changes in the performance of male genotypes with different female genotypes provide a “footprint” for the potential operation of intersexual coevolution [10].

## Methods

### 1. Isofemale lines and collecting experimental flies

To test for an interaction between male and female genotypes, we used a subset of the “worldwide” isofemale lines described previously [16]. Briefly, 10 lines each were originally created from five populations of *D. melanogaster* collected in Ithaca (New York), the Netherlands, Zimbabwe, Beijing (China) and Tasmania. Each line was then made isogenic (and hence a single genotype) by undergoing full-sibling mating for 12 generations. These lines were provided to us by A.G. Clark (Cornell University) in November 2008, and have since been maintained at 25°C on a two-week culture cycle with a 12 h light ∶ 12 h dark photoperiod in 25 mm diameter vials with standard cornmeal/molasses/killed-yeast medium. All experimental replicates were conducted under the same conditions.

We previously screened males from these 50 worldwide lines for their ability to induce maternal investment in a population of isogenic, outbred females (derived from flies originally collected on the west coast of North America), and found substantial genetic variation within populations (but not between populations) for this trait [16]. For the present study, we selected the best-performing male genotype (in terms of consistent male induction of maternal investment) from each geographical location (Ithaca: line I6, Netherlands: line N17, Zimbabwe: line Z23, Beijing: line B10, Tasmania: line T7). We used the best-performing genotype from each location because they were the least likely to have had their performance reduced by the inbreeding used to make the lines isogenic. Additional analysis of data from our previous study [16] confirms that males from these five isofemale lines did not differ in their ability to stimulate reproductive investment in the isogenic females used for that study (F_4,15_ = 0.90, p = 0.49). We next determined whether the relative performance of these five male genotypes varied depending on female genotype. We mated males from each of the five isofemale lines to females from each of the same five isofemale lines in all possible combinations to create a 5×5 matrix (25 combinations in total). We set up 10 experimental replicates in 5 blocks (2 replicates per block, performed on subsequent weeks), and surveyed all 25 combinations in each replicate. The methods described below are for a single replicate.

To collect experimental flies, we set up 10 vials for each isofemale line containing food medium with live yeast added to the surface (to stimulate fecundity) and 10–20 pairs of flies per vial. After three days, the flies were transferred into fresh vials containing medium and live yeast for an additional two days before they were discarded. We visually regulated larval density in both sets of vials 2–3 days after egg deposition by removing larvae from any vials that appeared overcrowded, resulting in a density of 150–200 larvae per vial. From the first set of vials we collected 70 males per line 13 days after oviposition, and from the second set of vials we collected 45–50 virgin females per line 9–10 days after oviposition. The males and the females were held separately in groups of 10–20 in vials containing food medium for 3–4 days until the experiments began.

### 2. Measuring mating speed and female reproductive investment

We began each replicate by setting up a series of mating observation vials containing a small amount of food medium and a cardstock paper divider that vertically separated the vial into two halves. Using light CO_2_ anesthetization, a single female was placed on one side of the divider, and two males (both from the same isofemale line) were placed on the other. As discussed above, females were either combined with males from their own isofemale line or with males from one of four other isofemale lines (originating from four different continents). A foam plug was then pushed into the vial to keep the two halves separate. These vials were set up 24 hours before the experiments began to allow the flies time to recover from CO_2_ anesthetization before mating trials.

Within each replicate, we set up seven mating observation vials for each male-female combination (175 vials in total). Mating observations began 3 h after lights-on at room temperature by gently lifting the foam plug so that the males and females could interact. We observed all vials for signs of mating over a 4 h period, and measured mating speed as the time at which five (out of seven total) females had begun mating for a single combination. Some male-female combinations did not result in the successful mating of five females during this period. For these combinations, we assigned a mating speed of 240 minutes (the maximum time allowed), regardless of the number of matings that had occurred.

We then measured female reproductive investment as in our previous study [16]. When five females from a specific combination had finished mating, we transferred them individually into 17 mm diameter oviposition test tubes (with a scored surface to promote oviposition) for 22 h, and then transferred them into fresh oviposition test tubes with a scored surface for an additional 22 h. The numbers of eggs laid by each female were counted for both sets of test tubes; the egg counts from the first set were recorded as “day-1 fecundity”, and the egg counts from the second set were recorded as “day-2 fecundity”.

At the end of the second 22 h period (hours 23–44), we combined the five mated females from a single male-female combination into an egg-laying chamber that contained a Petri dish filled with food medium. The females were allowed to oviposit on this dish for 4 h, at which time they were discarded. For each dish (i.e. each combination), we arranged 10 eggs (when possible) on their dorsal side and photographed them using an Olympus MicroFire digital camera and PictureFrame 2.0 software. We used ImageJ software (version 1.43u) to measure egg volume (*V*) with the formula for a prolate spheroid, 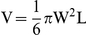, where *W* is the length of the equatorial diameter, and *L* is the length of the polar axis. Eggs were measured at the end of the second day following mating because previous work from our lab indicated that male-mediated effects on egg volume cannot be detected before this time (A.D. Stewart, T.A.F. Long and W.R. Rice, unpublished data).

Finally, we calculated female total reproductive investment as in our previous study [16], by multiplying the mean day-2 fecundity by the mean egg volume for each male-female combination. We used day-2 fecundity for this metric because our previous study found a significant effect of male genotype on day-2 fecundity, but not on day-1 fecundity (consistent with male effects on egg volume) [16]. Since we are interested in male influences on female reproductive investment, the most appropriate metrics to include for this trait are those affected by male genotype (day-2 fecundity and egg volume, measured immediately after day-2 fecundity).

### 3. Data Analysis

We tested for a male-by-female interaction for mating speed, day-1 fecundity, day-2 fecundity, egg volume and total reproductive investment using a multifactor random effects Analysis of Variance (ANOVA). Because our experimental design was balanced across cells, we used the Expected Mean Squares approach, with “Experimental Block”, “Male Genotype”, “Female Genotype” and all possible interactions as main, random factors. We also used the Restricted Maximum Likelihood approach to obtain variance component estimates (and 95% confidence intervals for those estimates) for the male×female interaction terms. For analyses of mating speed, the unit of replication was the time at which five females had begun mating within a male-female combination, and for all analyses of fecundity, the unit of replication was the mean fecundity of these five mated females. For measurements of egg volume we analyzed the mean egg volume produced by each male-female combination, and for reproductive investment we measured the product of mean day-2 fecundity and mean egg volume.

## Results

### 1. Male-by-female interaction for mating speed

**Figure 1:**
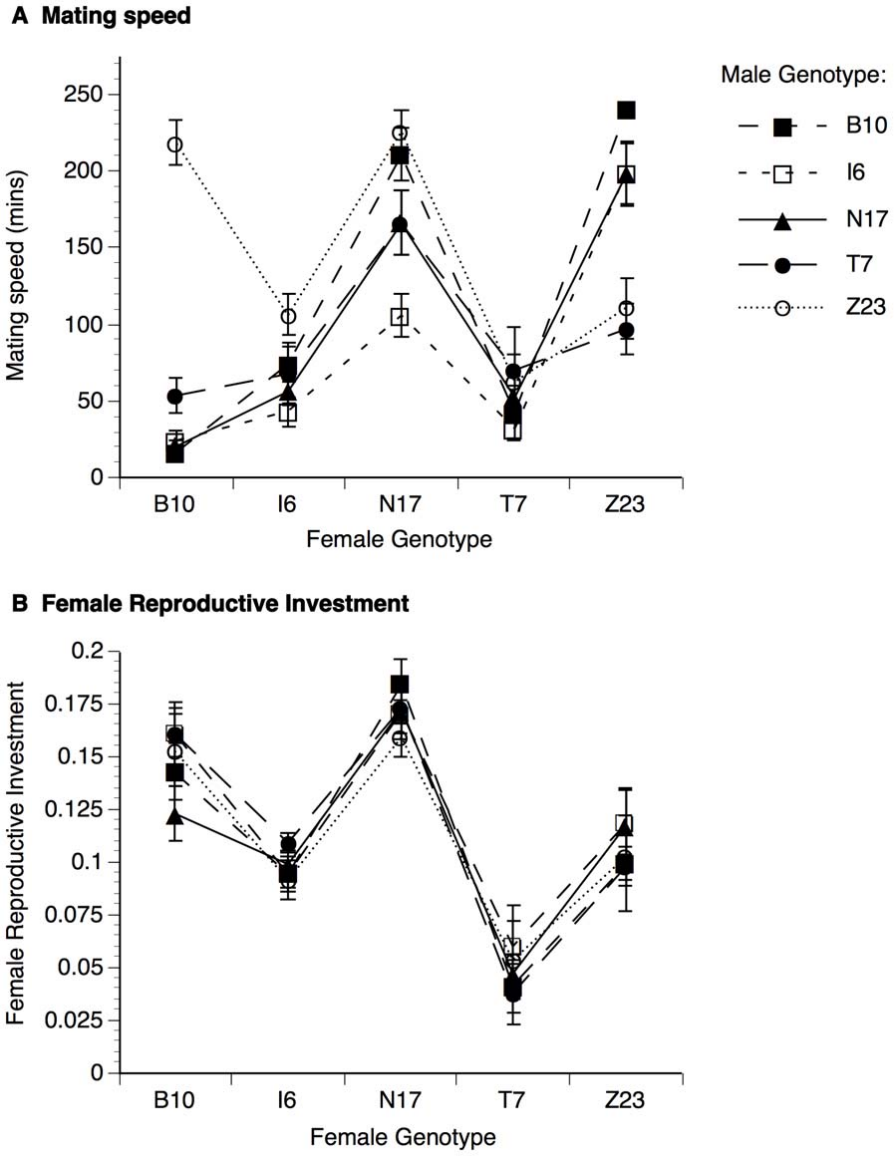
Mean mating speed and female reproductive investment for all combinations of male and female genotypes. (A) For mating speed, the interaction between male and female genotypes is evident from the crossing pattern of lines representing different male genotypes. (B) For female reproductive investment, these lines run approximately parallel one another, indicating there is no interaction between male and female genotypes. Error bars indicate standard errors.

Mating speed was strongly influenced by female genotype (Table 1A). Although experiment block interacted significantly with female genotype, there was not a significant block×female×male interaction for this trait. Most importantly, we found a strong male-by-female crossing interaction for mating speed (Table 1A; Figure 1A), which accounted for 31.8% of the variation in this trait (95% confidence interval = 8.1%, 55.4%). This latter result indicates that the relative mating speed of males was strongly dependent on the genotype of the female that they were courting. These results remained significant even when the Zimbabwe line was removed from the analysis (male genotype: F_3,116_ = 2.79, p = 0.12; female genotype: F_3,116_ = 16.95, p<0.0001; male-by-female interaction: F_9,116_ = 2.69, p = 0.0071).

**Table 1:** Analysis of variance testing the effects of experimental block, male genotype, female genotype and their interactions on (A) mating speed and (B) female reproductive investment.

(A) Mating speed			
Effect	d.f.	*F*	*p*
Experimental Block	4, 124	2.83	0.0650
Female Genotype	4, 124	5.87	0.0029*
Experimental Block×Female Genotype	16, 124	2.62	0.0010*
Male Genotype	4, 124	1.15	0.3702
Experimental Block×Male Genotype	16, 124	1.04	0.4129
Female Genotype×Male Genotype	16, 124	14.10	<0.0001*

(B) Female reproductive investment			
Effect	d.f.	*F*	*p*
Experimental Block	4, 151	5.33	0.0069*
Female Genotype	4, 151	30.69	<0.0001*
Experimental Block×Female Genotype	16, 151	2.12	0.0102*
Male Genotype	4, 151	0.89	0.5047
Experimental Block×Male Genotype	16, 151	0.99	0.4666
Female Genotype×Male Genotype	16, 151	0.97	0.4965

Experimental block, male genotype, female genotype and their interactions were treated as random effects. All three-way interaction terms (Experimental Block×Male Genotype×Female Genotype) were non-significant with p>0.50.

### 2. No male-by-female interaction for maternal investment

Both day-1 and day-2 fecundity were strongly affected by female genotype (day-1 fecundity: F_4,188_ = 37.18, p<0.0001; day-2 fecundity: F_4,188_ = 47.87, p<0.0001). There was no effect of male genotype on day-1 fecundity (F_4,188_ = 2.23, p = 0.14), or day-2 fecundity (F_4,188_ = 1.36, p = 0.35), and we did not detect a male-by-female interaction for fecundity on either day (day-1 fecundity: F_16,188_ = 1.48, p = 0.11; day-2 fecundity: F_16,188_ = 1.19, p = 0.28). Similarly, there was a strong effect of female genotype on egg volume (F_4,151_ = 299.67, p<0.0001), but there was no variation associated with male genotype (F_4,151_ = 0.56, p = 0.70), nor was there a male-by-female interaction for this trait (F_16,151_ = 0.95, p = 0.51). Finally, we found significant variation associated with experimental block and female genotype (and their interaction) for the amount of female reproductive investment (day-2 fecundity×egg volume), but there was no effect of male genotype on this trait (Table 1B). We did not detect a male-by-female interaction for female reproductive investment (Table 1B; Figure 1B), as this interaction only accounted for 0.3% of the variation in this trait (95% confidence interval = −2.3%, 2.9%). This indicates that the ability of a male genotype to influence his mate's investment in reproduction did not depend on her genotype.

## Discussion

In this study, we set out to identify the potential for intersexual coevolution in cosmopolitan populations of *D. melanogaster*. We compared genotypes from geographically distant populations for two traits associated with male reproductive success: mating speed (a pre-copulatory trait) and female reproductive investment (a post-copulatory trait). By crossing males and females from different populations, we can reveal the “footprint” of this coevolution: a male-female interaction for the expression of a trait [10]. We previously demonstrated substantial genetic variation in male ability to influence reproductive investment in their mates [16], and genetic variation in mating speed has been documented in both male and female *D. melanogaster* [13]. Here we tested whether variation in these two traits has the potential to result in intersexual coevolution, as indicated by a male-female genotype interaction.

We found a strong effect of female genotype, but not male genotype, on mating speed (Table 1A), indicating intrinsic variation among females in how rapidly they mate. Although males from these lines do not appear to differ intrinsically in their mating speed, there was a strong male-female interaction for this trait (Table 1A, Figure 1A), indicating that the effectiveness of male courtship varies depending on the genotype of the female being courted, and suggesting the potential for intersexual coevolution for this pre-copulatory trait. Without more detailed behavioral observations, however, it is difficult to speculate whether females and/or males are responsible for this interaction. For example, mating speed may be primarily determined by female mating preferences, with these preferences varying according to the female genotype. This would be consistent with the observation that the mating isolation between Zimbabwe females and cosmopolitan males appears to be driven by female choice, since cosmopolitan males court Zimbabwe females normally [14]. Alternatively (or additionally), mating speed may be determined by male courtship intensity, which could change in a male-specific manner based on some perceived index of female quality. Such changes in courtship intensity have been reported in male *D. melanogaster* in response to changes in female cuticular hydrocarbon profiles [18] and female body size [19].

Both males and females from the Zimbabwe line contributed strongly to the male-female interaction for mating speed (Figure 1A). This result was not unexpected, as there is evidence that the Zimbabwe population is genetically differentiated from other cosmopolitan populations of *D. melanogaster* [20], and Zimbabwe females prefer to mate with males from their own population [14], as we found here. What was surprising, however, was the particularly strong pre-copulatory reproductive isolation between our Zimbabwe and Beijing genotypes in both directions (i.e. Beijing females combined with Zimbabwe males and Zimbabwe females combined with Beijing males; Figure 1A). In fact, the majority of these combinations did not result in 5 successful matings out of the 7 surveyed pairs observed over a 4 h period (and were hence assigned a mating speed of 240 minutes, as described above). Over 10 experimental replicates, the combination of Beijing females with Zimbabwe males resulted in an average of only 2.8 successful matings out of 7 pairs, and the combination of Zimbabwe females with Beijing males resulted in an average of only 1.3 successful matings out of 7 pairs. While the Zimbabwe female preference to mate with males from their own population (and avoid mating males from other populations) is well established, it is unusual for females from other cosmopolitan populations of *D. melanogaster* to exhibit a strong aversion to Zimbabwe males [14], [15].

Another interesting result was the strong male-female interaction for mating speed that persisted even when we removed the Zimbabwe line from our analysis. With the exception of those from southern Africa, cosmopolitan populations of *D. melanogaster* are often assumed to have indiscriminate mating preferences [21] and are treated as one large population (referred to as the “M-type” [22]). However, there are an increasing number of studies demonstrating male-female genotype interactions for mating speed between *D. melanogaster* populations (in addition to evidence for such interactions within populations [23], [24]). For example, male-female interactions for mating speed have been reported among populations collected in Amherst, Novosibirsk and the Pacific [25], between Canton-S and populations from West Africa [26], and between populations collected from opposite slopes of “Evolution Canyon” in Israel [27]. The strong male-female interaction for mating speed that we observed among genotypes collected from Beijing, Tasmania, the Netherlands and Ithaca builds upon these previous studies to suggest that variation in mating preferences among *D. melanogaster* populations may be widespread.

Although we were able to detect a strong effect of female genotype on fecundity, egg size and total reproductive investment (Table 1B), there was no variation among the five male genotypes for any of these post-copulatory traits. These results were not surprising because *i*) we previously found no variation among populations for male effects on female reproductive investment [16], *ii*) we selected the best performing male genotypes for these traits from each of our five populations to use in this study, and *iii*) males from these five genotypes did not differ in their effect on female reproductive investment (using a controlled female genotype) in our previous study (see Methods). More importantly, we did not detect a male-female interaction for female reproductive investment (Figure 1B), or any of its components (fecundity or egg volume). This was somewhat unexpected, as both male and female reproductive proteins evolve rapidly in many species [28], and previous studies have identified male-female interactions for post-copulatory traits in houseflies [10], flour beetles [29], [30] and within and between populations of *Drosophila* [31]–[34]. One potential explanation is that we did not have sufficient statistical power to detect a male-female interaction for maternal investment. However, the 95% confidence interval for the variance component attributed to the male-female interaction was almost 10 times smaller for reproductive investment than for mating speed, indicating that we actually had more statistical power to detect a male-female interaction for our post-copulatory trait than for our pre-copulatory trait. Because male ability to influence female reproductive investment did not depend on the female genotype, it is possible that males and females do not coevolve with respect to this trait. Alternatively, any coevolution between the sexes may occur at such a slow rate that it is masked at any single point in time. Indeed, reproductive isolation between wild populations of *D. melanogaster* appears to evolve more rapidly via pre-copulatory sexual behaviors than post-copulatory phenotypes [14], suggesting that any coevolution between the sexes with respect to maternal investment may occur at a much slower rate than that for mating speed.

It is important to consider how using a single genotype from each geographic location could influence our results. Using the male genotypes that were most effective at stimulating reproductive investment in their mates should increase our experimental power to detect a male- by-female interaction for this trait, since these males are likely to be more successful in sexual selection and/or sexual conflict compared to less effective male genotypes that may be lower quality in general. These genotypes should also have the lowest influence of any inbreeding that accrued when making each worldwide line isogenic. Although we did not detect a male-female genotype interaction for female reproductive investment among our five selected genotypes, we found a very strong interaction for mating speed among the same genotypes. An important consideration, however, is that we only sampled one genotype from each of five geographic locations. A larger diversity of genotypes or populations may have improved our study, but the geometric increase in sample size required to survey all pair-wise crosses precluded this option. This study could also be replicated using outbred populations (as opposed to isofemale lines) from several different geographic locations. Although this design would add experimental noise and reduce our ability to detect a male-female interaction, it would allow us to generalize our results beyond single genotypes. It is possible that the genotypes we selected were not true representations of the populations they originated from. In our study we found a highly significant male-female interaction for mating speed that persisted without the Zimbabwe line and evidence for strong behavioral reproductive isolation between the Zimbabwe and Beijing lines. Before we can make general conclusions about the implications of these findings, we need to incorporate data from additional genotypes and/or populations.

Although the male-female interaction we found for mating speed among our five populations indicates the potential for these populations to undergo sexual coevolution, this is not the only possible explanation for this interaction. For example, it is possible that the observed differences in mating speed between populations are the result of stochastic processes, such as founder effects and/or genetic drift, causing the rank-order of specific genotype combinations to be largely random. In addition, mating speed may reflect female mate preferences, but the male-female interaction for this trait would not result in coevolution unless rapid mating is associated with an increase or decrease in female fitness, which has not been investigated. This pattern could also be a genetic consequence of the speciation process [14], [20], [35]. To ultimately confirm that our observed male-female interaction reflects sexual coevolution, we would have to demonstrate that changes in a male (or female) trait associated with mating speed elicits evolutionary changes in a comparable female (or male) trait.

Here, we identified a male-female interaction for a pre-copulatory trait (mating speed), but not a post-copulatory trait (female reproductive investment) in *D. melanogaster*. Our findings support the hypothesis that coevolution for pre-copulatory mating interactions may be ongoing in this species, but the nature of our experimental design is unable to exclude alternate explanations. Instead, our study is intended to motivate additional studies into the role of pre-copulatory sexual coevolution in cosmopolitan populations of *D. melanogaster*. Studies of sexual coevolution in this species often focus on post-copulatory traits (e.g. [31], [32]), but the fact that we detected a male-female interaction for mating speed and not female reproductive investment suggests that the potential for sexual coevolution to influence mating preferences in *D. melanogaster* warrants further investigation.

## References

[1] Ferveur JF (2005). Cuticular hydrocarbons: their evolution and roles in *Drosophila* pheromonal communication.. Behav Genet.

[2] Hosken DJ, Stockley P (2004). Sexual selection and genital evolution.. Trends Ecol Evol.

[3] Panhuis TM, Butlin R, Zuk M, Tregenza T (2001). Sexual selection and speciation.. Trends Ecol Evol.

[4] Parker GA, Blum MS, Blum NA (1979). Sexual selection and sexual conflict.. Sexual Selection and Reproductive Competition in Insects.

[5] Rice WR (1996). Sexually antagonistic male adaptation triggered by experimental arrest of female evolution.. Nature.

[6] Gavrilets S (2000). Rapid evolution of reproductive barriers driven by sexual conflict.. Nature.

[7] Parker GA, Partridge L (1998). Sexual conflict and speciation.. Philos Trans R Soc Lond, Ser B: Biol Sci.

[8] Rice WR, Howard DJ, Berlocher SH (1998). Intergenomic conflict, interlocus antagonistic coevolution, and the evolution of reproductive isolation.. Endless Forms: Species and Speciation.

[9] Arnqvist G, Edvardsson M, Friberg U, Nilsson T (2000). Sexual conflict promotes speciation in insects.. Proc Natl Acad Sci USA.

[10] Andrés JA, Arnqvist G (2001). Genetic divergence of the seminal signal—receptor system in houseflies: the footprints of sexually antagonistic coevolution?. Proc R Soc Lond, Ser B: Biol Sci.

[11] Rowe L, Cameron E, Day T (2003). Detecting sexually antagonistic coevolution with population crosses.. Proc R Soc Lond, Ser B: Biol Sci.

[12] Fulker DW (1966). Mating speed in male *Drosophila melanogaster*: a psychogenetic analysis.. Science.

[13] Mackay TFC, Heinsohn SL, Lyman RF, Moehring AJ, Morgan TJ (2005). Genetics and genomics of *Drosophila* mating behavior.. Proc Natl Acad Sci USA.

[14] Wu CI, Hollocher H, Begun DJ, Aquadro CF, Xu Y (1995). Sexual isolation in *Drosophila melanogaster*: a possible case of incipient speciation.. Proc Natl Acad Sci USA.

[15] Ting CT, Takahashi A, Wu CI (2001). Incipient speciation by sexual isolation in *Drosophila*: concurrent evolution at multiple loci.. Proc Natl Acad Sci USA.

[16] Pischedda A, Stewart AD, Little MK, Rice WR (2011). Male genotype influences female reproductive investment in *Drosophila melanogaster*.. Proc R Soc Lond, Ser B: Biol Sci.

[17] Sheldon BC (2000). Differential allocation: tests, mechanisms and implications.. Trends Ecol Evol.

[18] Billeter JC, Atallah J, Krupp JJ, Millar JG, Levine JD (2009). Specialized cells tag sexual and species identity in *Drosophila melanogaster*.. Nature.

[19] Long TAF, Pischedda A, Stewart AD, Rice WR (2009). A cost of sexual attractiveness to high-fitness females.. PLoS Biol.

[20] Begun DJ, Aquadro CF (1993). African and North American populations of *Drosophila melanogaster* are very different at the DNA level.. Nature.

[21] Henderson NR, Lambert DM (1982). No significant deviation from random mating of worldwide populations of *Drosophila melanogaster*.. Nature.

[22] Hollocher H, Ting CT, Wu ML, Wu CI (1997). Incipient speciation by sexual isolation in *Drosophila melanogaster*: extensive genetic divergence without reinforcement.. Genetics.

[23] Casares P, Carracedo MC, Miguel ES, Piñeiro R, Garcia-Florez L (1993). Male mating speed in *Drosophila melanogaster*: Differences in genetic architecture and in relative performance according to female genotype.. Behav Genet.

[24] Welbergen P, Spruijt BM, Van Dijken FR (1992). Mating speed and the interplay between female and male courtship responses in *Drosophila melanogaster* (Diptera: Drosophilidae).. J Insect Behav.

[25] Connolly K, Burnet B, Kearney M, Eastwood L (1974). Mating speed and courtship behaviour of inbred strains of *Drosophila melanogaster*.. Behaviour.

[26] Scott D (1994). Genetic variation for female mate discrimination in *Drosophila melanogaster*.. Evolution.

[27] Iliadi K, Iliadi N, Rashkovetsky E, Minkov I, Nevo E (2001). Sexual and reproductive behaviour of *Drosophila melanogaster* from a microclimatically interslope differentiated population of ‘Evolution Canyon’ (Mount Carmel, Israel).. Proc R Soc Lond, Ser B: Biol Sci.

[28] Swanson WJ, Vacquier VD (2002). The rapid evolution of reproductive proteins.. Nat Rev Genet.

[29] Nilsson T, Fricke C, Arnqvist G (2002). Patterns of divergence in the effects of mating on female reproductive performance in flour beetles.. Evolution.

[30] Nilsson T, Fricke C, Arnqvist G (2003). The effects of male and female genotype on variance in male fertilization success in the red flour beetle (*Tribolium castaneum*).. Behav Ecol Sociobiol.

[31] Clark AG, Begun DJ, Prout T (1999). Female×male interactions in *Drosophila* sperm competition.. Science.

[32] Knowles LL, Markow TA (2001). Sexually antagonistic coevolution of a postmating-prezygotic reproductive character in desert *Drosophila*.. Proc Natl Acad Sci USA.

[33] Pitnick S, Miller GT, Schneider K, Markow TA (2003). Ejaculate-female coevolution in *Drosophila mojavensis*.. Proc R Soc Lond, Ser B: Biol Sci.

[34] Long TAF, Montgomerie R, Chippindale AK (2006). Quantifying the gender load: can population crosses reveal interlocus sexual conflict?. Philos Trans R Soc Lond, Ser B: Biol Sci.

[35] Alipaz JA, Wu C, Karr TL (2001). Gametic incompatibilities between races of *Drosophila melanogaster*.. Proc R Soc Lond, Ser B: Biol Sci.

